# Short-Term Results Suggest That Sleeved Stomach without Resection Is as Effective as Sleeve Gastrectomy in Improving Glucose Control in Type 2 Diabetes Mellitus Sprague-Dawley Rat Model

**DOI:** 10.1155/2020/9024923

**Published:** 2020-05-01

**Authors:** Wenzhuo Zhang, Jason Widjaja, Libin Yao, Yong Shao, Xiaocheng Zhu, Chao Li

**Affiliations:** ^1^Department of General Surgery, The Affiliated Hospital of Xuzhou Medical University, Xuzhou Jiangsu 221002, China; ^2^Institute of Digestive Diseases, Xuzhou Medical University, Xuzhou Jiangsu 221002, China

## Abstract

**Background:**

Although sleeve gastrectomy results in good weight loss and metabolic improvements, it is an irreversible procedure. Therefore, we attempted to assess the possibility of creating a sleeved stomach without resection. *Material and Methods*. A total of 22 male Sprague-Dawley rats with type 2 diabetes were randomly assigned into 3 different groups: (1) sleeve gastroplasty with gastric remnant-jejunal anastomosis (SGP, *n* = 8); (2) sleeve gastrectomy (SG, *n* = 8); and (3) SHAM (*n* = 6). Body weight, food intake, fasting blood glucose (FBG), hormonal analysis, and oral glucose tolerance test (OGTT) were performed and measured preoperatively and postoperatively.

**Results:**

During the postoperative period, SGP and SG showed significantly lower food intake and body weight when compared with the preoperative levels, respectively (*p* value < 0.05). Postoperatively, SGP and SG showed improvements in FBG and glucose tolerance levels compared to their respective preoperative levels (*p* < 0.05). FBG and glucose tolerance levels did not differ between SGP and SG postoperatively. SG resulted in a reduction in fasting ghrelin levels when compared with the preoperative level (*p* < 0.05). Fasting insulin levels did not differ preoperatively and postoperatively among all groups. Postoperatively, fasting GLP-1 levels were higher in SGP and SG when compared with the preoperative levels, but no statistical significance was observed. Compared preoperatively, the SGP and SG procedures resulted in a decline in HOMA-IR at postoperative 6th week (*p* < 0.05).

**Conclusion:**

Our animal experiment suggested that at least in the short term, sleeved stomach without resection resulted in similar weight loss and improved glucose control effects compared to sleeve gastrectomy.

## 1. Background

Sleeve gastrectomy (SG) has become the most performed procedure in bariatric surgery [1]. Weight loss and metabolic profile improvements after SG were comparable to the standard bariatric procedure in Roux-en-Y gastric bypass (RYGB) [2–5]. Furthermore, SG has a lower postoperative complication risk when compared to RYGB [6–8].

One of the disadvantages of SG is its irreversibility. On the other hand, RYGB, though with some extent of difficulty, is reversible. If one was to perform a “reversible” SG modification, it had to have at least a similar efficacy as SG. Thus, through animal experiments, we attempted to test the outcomes of performing a reversible sleeved stomach without resection. In this experiment, we used a suturing technique to create a sleeved stomach without resection and compared the weight loss and glucose control outcomes to SG.

## 2. Material and Methods

### 2.1. Animals

This study was approved by the ethics committee of the Xuzhou Medical University Research Animal Centre. All applicable institutional and national guidelines of China for the care and use of animals were followed. Eight- to ten-week-old male Sprague-Dawley (SD) rats were purchased from the Xuzhou Medical University Research Animal Centre. Constant temperature and humidity with 12 h day/12 h night cycle were maintained throughout the study. The type 2 diabetes model was induced through a high-fat diet and intraperitoneal injection of low-dose streptozotocin (STZ, 35 mg/kg) [9]. Random blood glucose levels were measured 72 h after STZ injection with a handheld glucometer. Rats with a random blood glucose level of >16.0 mmol/L in three consecutive days were considered to be diabetic.

### 2.2. Study Design

Our sleeved stomach without resection procedure was termed sleeve gastroplasty (SGP). Twenty-two diabetic male SD rats were randomly assigned into three different groups: (1) SGP with gastric remnant-jejunal anastomosis (SGP, *n* = 8); (2) SG (*n* = 8); and (3) SHAM (*n* = 6). Body weight, food intake, and fasting blood glucose (FBG) levels were preoperatively and postoperatively measured at the 2nd, 4th, and 6th week. OGTT was performed preoperatively and at postoperative 6th week. Fasting blood samples were taken preoperatively and at postoperative 6th week for hormonal analysis (ghrelin, insulin, and GLP-1).

### 2.3. SGP Procedure

**Figure 1:**
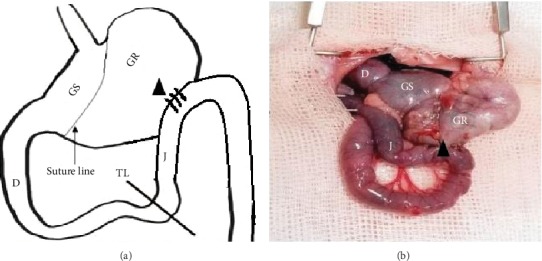
Surgical procedure for sleeve gastroplasty (SGP). (a) Drawn illustration of the SGP procedure. (b) Oral gavage with methylene blue at postoperative 6th week in the SGP model. GS: gastric sleeve; GR: gastric remnant; D: duodenum; J: jejunum; TL: Treitz ligament; triangle: gastric remnant.

To perform the SGP procedure, the stomach was sutured using a nonabsorbable 2-0 Mersilk suture (Figure 1). The stomach was sutured beginning from the angle and ended approximately 0.5 cm away from the pylorus, twice (two suture lines). It took approximately 4–5 sutures (each bite ~1–1.5 cm, simple interrupted manner) to complete one suture line. The suture was carefully performed to prevent injury of the short gastric and gastroepiploic vessels. Intraoperatively and at the end of the study (postoperative 6th week), methylene blue gavage was introduced to confirm that there was no communication between the two gastric pouches. Due to the possibility of gastric remnant dilation, additional anastomosis of the gastric remnant with jejunum, located approximately 5 cm distal to the ligament of Treitz, was created.

### 2.4. SG Procedure

Our SG procedure was performed using 4-0 nonabsorbable silk sutures and according to the method of Al-Sabah et al. [10]. Overall, the SG procedure resulted in the resection of the greater curvature from the distal antrum (approximately 0.5 cm proximal to the pylorus) until the angle of His, resecting the fundus completely [11, 12].

### 2.5. SHAM Procedure

For the SHAM group, a small incision was made on the anterior part of the stomach and closed to mimic the traumatic manipulation of the stomach. The abdomen was inspected for bleeding and continuously closed in layer with a 2-0 Mersilk suture.

### 2.6. OGTT and Blood Sample Collection

The 2-hour oral glucose tolerance test (OGTT) with 50% glucose solution gavage (3 mg/kg) was performed preoperatively and at postoperative 6th week in all groups. The blood glucose was measured from the tail vein at 0, 30, 60, 90, and 120 min using a handheld glucometer (approximately 1 *μ*L of blood samples was obtained per sampling) in the conscious rats.

During retro orbital blood sampling, the rats were anaesthetized with isoflurane using an “open-drop” method with cotton as the absorbent [13]. Fasting retro orbital blood samples were taken from overnight fasted rats preoperatively and at postoperative 6th week in all groups (approximately 1 mL of blood samples was obtained per sampling), followed by centrifugation (3,000 rpm for 10 min) to collect the plasma and stored at -80°C until further use. Hormonal analysis was performed using an enzyme-linked immunosorbent assay kit (ELISA kit, Shanghai Jianglai Industrial Limited By Share Ltd). Insulin resistance (HOMA-IR) was calculated using the homeostasis model assessment (HOMA), at preoperative and postoperative 6th week (HOMA‐IR = fasting insulin (mU/L) × fasting glucose (mmol/L)/22.5).

### 2.7. Statistical Analysis

All data are presented as mean ± standard deviation (SD). The area under the curve (AUC) was calculated using the trapezoidal method (GraphPad Prism 7). One-way ANOVA was used to assess differences among groups. Student's *t*-test was used to compare differences between means. All tests were two-tailed and considered statistically significant with *p* < 0.05.

## 3. Results

### 3.1. Body Weight and Food Intake

**Figure 2:**
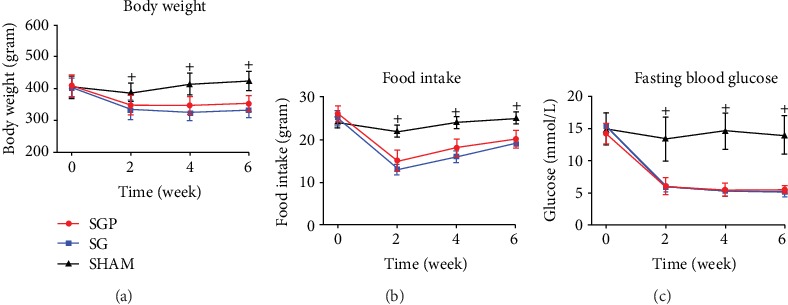
Change in (a) body weight, (b) food intake, and (c) fasting blood glucose. All data are presented as the mean ± standard deviation. ^+^Significant compared with SHAM (*p* < 0.05).

There were no significant differences in body weight or food intake among all groups preoperatively. Postoperatively, SGP and SG showed significantly lower food intake and body weight when compared with the preoperative levels, respectively (*p* < 0.05). At postoperative 6th week, there were no significant differences among SGP and SG in mean total body weight loss (13.3 ± 4.5 and 17.3 ± 4.6%, respectively, from preoperative body weight) and food intake reduction (23.5 ± 4.9 and 24.2 ± 3.6%, respectively, from preoperative food intake) (Figures 2(a) and 2(b)).

### 3.2. FBG and OGTT

**Figure 3:**
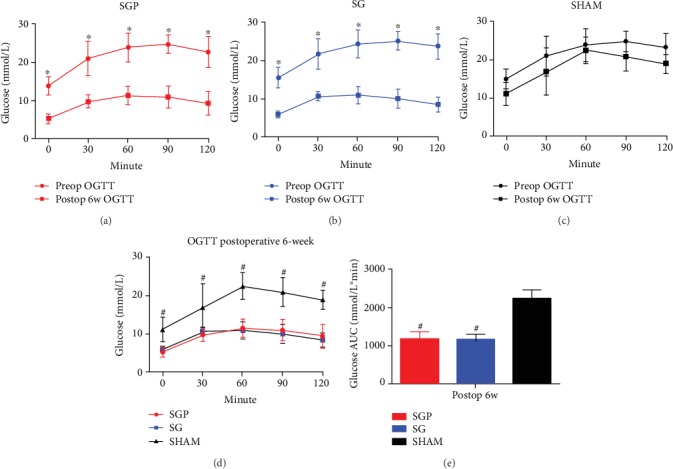
OGTT results preoperatively (preoperative) and postoperatively (postoperative): (a) SGP, (b) sleeve gastrectomy (SG), and (c) SHAM. Group comparison at postoperative level: (d) OGTT. (e) Glucose AUC. ^∗^ indicates significant difference (*p* value < 0.05). ^#^Significant compared with SHAM (*p* < 0.05).

At postoperative 6th week, FBG levels significantly declined from the preoperative level by 60.6 ± 7.2% and 64.7 ± 10.7% in the SGP and SG groups, respectively (*p* < 0.05) (Figure 2(c)). Moreover, at postoperative 6th week, glucose tolerance (through OGTT results) significantly improved in the SGP and SG groups when compared with the preoperative levels, respectively (*p* < 0.05) (Figures 3(a) and 3(b)). The glucose AUC at postoperative 6th week did not significantly differ between the SGP and SG groups (Figure 3(e)). The SHAM group preoperative and postoperative results remained at similar levels.

### 3.3. Hormonal Analysis and HOMA-IR

**Figure 4:**
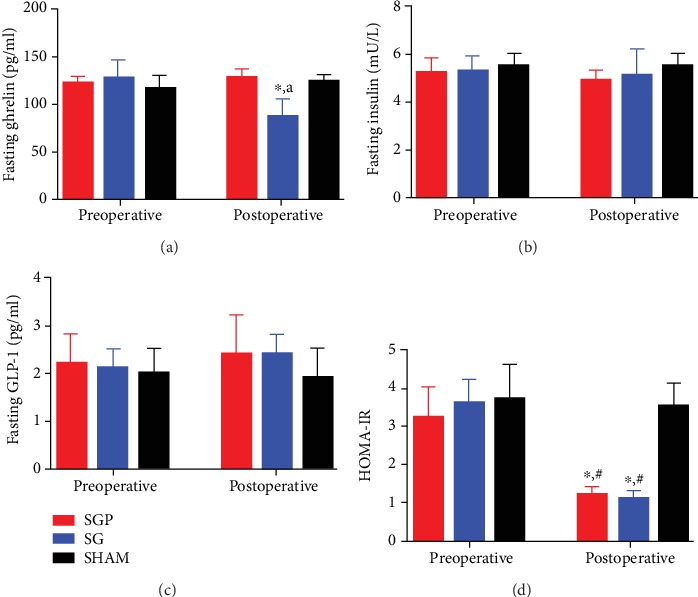
Group comparison preoperative and postoperative 6-week: (a) fasting ghrelin, (b) fasting insulin, (c) fasting GLP-1, and (d) HOMA-IR. ^∗^Significant compared with its respective preoperative level (*p* < 0.05). ^#^Significant compared with postoperative SHAM (*p* < 0.05). ^a^Significant postoperative SG compared with postoperative SGP and SHAM (*p* < 0.05).

The SG procedure resulted in a significant reduction in fasting ghrelin levels when compared with the preoperative level (*p* value < 0.05) (Figure 4(a)). Fasting insulin levels did not significantly differ preoperatively and postoperatively among all groups (Figure 4(b)). Fasting GLP-1 levels were higher postoperatively in SGP and SG than in preoperative levels, but no statistical significance was observed (Figure 4(c)). Compared preoperatively, SGP and SG procedures resulted in a significant decline in HOMA-IR at postoperative 6th week (*p* < 0.05). HOMA-IR at postoperative 6th week did not significantly differ between the SGP and SG (Figure 4(d)).

## 4. Discussion

Our short-term (6 weeks) animal experiment showed that compared to SG, sleeved stomach without resection procedure (SGP) resulted in similar weight loss and glucose control improvements.

Postoperatively, SGP and SG showed similar improvements in FBG, OGTT, and HOMA-IR levels when compared to their preoperative levels. The gastric pouch for food transit in the SGP model was similarly altered as in the SG model. It was previously reported that rapid gastric emptying and accelerated intestinal transit could be the key factors for glucose control improvements following SG procedures [14, 15]. If this hypothesis is true, the SGP model should have a similar diabetes remission mechanism as SG.

SG and our SGP procedure resulted in significant improvements in glucose control [2]. Furthermore, it was reported that performing only duodenal exclusion (duodenal-jejunal bypass, DJB), without bypassing the stomach, did not yield significant glucose control [16]. This evidence suggests that the gastric system might have a significant impact on glucose homeostasis.

Evidence on the importance of the stomach in improving glucose control is abundant, although it is still lacking in clear definition. A recent study reported that gastric banding and gastric banded plication, procedures that modified only the stomach, resulted in excellent weight loss and improvements in metabolic parameters [17]. As the stomach is an organ for digestion [18], bypassing the majority of the stomach might result in expedited undigested nutrients to the intestine, which is crucial for initiating the hindgut theory [19]. This can be seen as a humbler means to elucidate the gastric effect on diabetes remission. Another possible mechanism could be that by bypassing the stomach, there might be some unrecognized cells or receptors in the gastric mucosa that play a crucial role in the glucose homeostasis control, which were rendered ineffective [20, 21]. Lastly, it is also possible that the “nonstimulated” gastric mucosa resulted in altered production and secretion levels of gastric hormones that are related to glucose homeostasis (gastrin, somatostatin, etc.) [22–24]. All of these studies suggest that future obesity and T2DM research should be emphasized in the stomach.

We believe that possible gastric remnant dilation could be a limitation in the sleeved stomach without a resection procedure. It has been reported that when the gastric remnant outlet is obstructed, gastric remnant dilation subsequently occurs [25, 26]. Therefore, we created an additional gastric remnant-jejunal anastomosis for the SGP group, which did not affect the glucose improvement outcome.

SG procedure resulted in not only a significant weight loss outcome but also diabetes remission efficacy. A recent study reported the SG outcomes as a metabolic surgery in inducing diabetes remission with body mass index < 30 kg/m^2^ [27]. It is crucial if one was to attempt to create a reversible SG modification to have a similar postoperative impact (not only for the sake of creating a reversible procedure). Hence, our experiment provides a premise that the sleeved stomach without resection procedure (SGP) might also have an impact on T2DM patients. Following this study, the next plausible focus should be to discover a safe material and/or method to recreate the SGP procedure in a larger animal model.

Our animal SGP experiment appeared to provide an antidiabetic effect to a similar degree as in SG. The SGP procedure might alternatively serve as a first-stage treatment for highly obese patients complicated with T2DM. Performing aggressive procedures such as duodenal switch in these obese patients might generate extra intraoperative difficulties and complications. Thus, the SGP model could *hypothetically* help (as a first-stage procedure) to gradually lower the patient's weight before performing a more aggressive procedure if or when it is needed.

Our study was limited by its sample size and short duration. We also acknowledge that our SGP model was performed using the suturing method (which might not be plausible in humans or larger animal models). In this experiment, the presence of gastric remnant-jejunum anastomosis was also noted, and currently, we cannot conclusively determine whether it plays an additional role in delivering the outcomes that were seen. Furthermore, we only analyzed the fasting levels of three hormones (ghrelin, insulin, and GLP-1). Nonetheless, the results were promising, and we ponder into the future to identify a safe material and/or method and perform SGP on larger animal models.

## 5. Conclusion

In the short term, our animal model showed that sleeved stomach without resection (sleeve gastroplasty) has weight reduction and diabetes-remitting potentials similar to the standard sleeve gastrectomy procedure. Identifying a safe material and/or method for SGP application in larger animal models should be the focus of future studies.

## Data Availability

The data sets produced and/or analyzed during this study are available from the corresponding author on request.
